# A Novel Model for Nephrotic Syndrome Reveals Associated Dysbiosis of the Gut Microbiome and Extramedullary Hematopoiesis

**DOI:** 10.3390/cells10061509

**Published:** 2021-06-15

**Authors:** Jasmin I. Maier, Manuel Rogg, Martin Helmstädter, Alena Sammarco, Gerd Walz, Martin Werner, Christoph Schell

**Affiliations:** 1 Institute of Surgical Pathology, Faculty of Medicine, Medical Center-University of Freiburg, 79106 Freiburg, Germany; jasmin.maier@uniklinik-freiburg.de (J.I.M.); manuel.rogg@uniklinik-freiburg.de (M.R.); alena.sammarco@uniklinik-freiburg.de (A.S.); martin.werner@uniklinik-freiburg.de (M.W.); 2 Department of Medicine IV, Faculty of Medicine, Medical Center-University of Freiburg, 79106 Freiburg, Germany; martin.helmstaedter@uniklinik-freiburg.de (M.H.); gerd.walz@uniklinik-freiburg.de (G.W.)

**Keywords:** nephrotic syndrome, focal segmental glomerulosclerosis, EPB41L5, gut microbiome, extramedullary hematopoiesis

## Abstract

Glomerular kidney disease causing nephrotic syndrome is a complex systemic disorder and is associated with significant morbidity in affected patient populations. Despite its clinical relevance, well-established models are largely missing to further elucidate the implications of uncontrolled urinary protein loss. To overcome this limitation, we generated a novel, inducible, podocyte-specific transgenic mouse model (Epb41l5^fl/fl^*Nphs1-rtTA-3G*tetOCre), developing nephrotic syndrome in adult mice. Animals were comprehensively characterized, including microbiome analysis and multiplexed immunofluorescence imaging. Induced knockout mice developed a phenotype consistent with focal segmental glomerular sclerosis (FSGS). Although these mice showed hallmark features of severe nephrotic syndrome (including proteinuria, hypoalbuminemia and dyslipidemia), they did not exhibit overt chronic kidney disease (CKD) phenotypes. Analysis of the gut microbiome demonstrated distinct dysbiosis and highly significant enrichment of the Alistipes genus. Moreover, *Epb41l5*-deficient mice developed marked organ pathologies, including extramedullary hematopoiesis of the spleen. Multiplex immunofluorescence imaging demonstrated red pulp macrophage proliferation and mTOR activation as driving factors of hematopoietic niche expansion. Thus, this novel mouse model for adult-onset nephrotic syndrome reveals the significant impact of proteinuria on extra-renal manifestations, demonstrating the versatility of this model for nephrotic syndrome-related research.

## 1. Introduction

Glomerular kidney disease associated with nephrotic syndrome (NS) results in significant morbidity in affected patient populations. The glomerular filtration barrier is a three-layered filtration unit composed of fenestrated endothelium, the glomerular basement membrane (GBM) and highly specialized epithelial cells (commonly termed as podocytes) [1,2]. Unhalted proteinuria ultimately results in chronic kidney disease (CKD) and related end stage renal disease (ESRD). A significant subset of glomerular diseases including focal segmental glomerulosclerosis (FSGS), minimal change disease (MCD) and membranous glomerulonephritis (MGN) are characterized by a clinical symptom complex collectively termed as nephrotic syndrome (NS).

NS is defined by the co-occurrence of proteinuria, hypoalbuminemia, dyslipidemia and edema [3]. The cardinal symptom of NS, namely overt proteinuria, is attributed to a dysfunction of the glomerular filtration barrier and related to direct or indirect podocyte damage [4,5,6]. Continuous proteinuria translates into multiple, systemic NS manifestations such as hypoproteinemia and dyslipidemia due to impaired lipoprotein clearance [7]. Moreover, loss of functional blood proteins such as hormones, hormone-binding proteins and anti-thrombotic factors impair multiple regulatory systems essential for body homeostasis [8,9].

Despite significant progress in the understanding of NS, organ-specific manifestations in the context of proteinuria are poorly defined. Detailed histopathological studies of NS without simultaneous renal failure are missing due to the progressive character of NS. Moreover, established experimental models recapitulating essential features of human NS exhibit several limitations [10,11,12]. Most targeted, transgenic murine models are based on the podocyte-specific expression of Cre-recombinase transgenes [13,14,15,16,17,18,19,20]. Due to prenatal Cre-expression, the majority of these models develop congenital or premature glomerular disease. Overlapping CKD phenotypes are frequently observed in these models, impairing the causative attribution of observed phenotypes and presentations of NS. Similar limitations are also evident for toxin-induced NS (e.g., nephron toxic serum (NTS) or doxorubicin) due to strain sensitivity, variance in response, systemic side effects and disease progression.

Here, we developed an inducible, podocyte-specific mouse model for nephrotic syndrome in adult mice. Inducible knockout of *Epb41l5* in adult mice (Epb41l5^fl/fl^*Nphs1-rtTA-3G*tetOCre) was characterized by hallmark features of severe NS, with minimal overlapping CKD. We utilized this novel mouse model to analyze the impact of NS on microbiome composition and organ pathologies, including extramedullary hematopoiesis of the spleen, demonstrating the versatility of this model for nephrotic syndrome-related research.

## 2. Materials and Methods

### 2.1. Animals

Inducible, podocyte-specific *Epb41l5* knockout (KO) mice (Epb41l5^fl/fl^*Nphs1-rtTA-3G*tetOCre) were generated using a previously described *Epb41l5^fl/fl^* allele [21] in combination with a podocyte-specific, inducible Cre line (*Nphs1-rtTA-3G* (syn. *NEFTA*)). The *NEFTA* transgene was kindly provided by Jeffrey H. Miner [22]. Mice were maintained on a 129/Sv-mixed background. Cre expression was induced in 6-week-old KO and control mice by application of 2 mg/mL doxycycline and 5% sucrose via drinking water for 7 days. Epb41l5^fl/fl^*Nphs1-rtTA-3G and Epb41l5^fl/+^*Nphs1-rtTA-3G*tetOCre littermates were doxycycline-induced in parallel and used as controls, respectively (referred to as wild type “WT”). Mice were sacrificed 4, 8 and 12 weeks after induction. All mouse experiments were performed according to the National Institutes of Health Guide for the Care and Use of Laboratory Animals, as well as the German law governing the welfare of animals. All studies were approved by the Regierungspräsidium Freiburg (G-17/127), Germany.

### 2.2. Cell Culture

Murine bone-marrow derived macrophages (MΦs) (J2 recombinant retrovirus-immortalized) were a generous gift from Philipp Henneke (Medical Center—University of Freiburg, Freiburg, Germany) and Douglas T. Golenbock (UMass Medical School, Worcester, USA [23]). Macrophages were cultured in DMEM supplemented with glutamine and 10% FBS at 37 °C, 95% air and 5% CO_2_. Macrophages were tested for mycoplasma contamination by PCR (Mycoplasma PCR Detection Kit; Hiss Diagnostics GmbH, Freiburg, Germany). For stimulation experiments, macrophages were seeded and cultured on Collagen IV-coated, eight-well polymer coverslips (Ibidi GmbH, Gräfelfing, Germany) for 24 h in normal growth medium. Thereafter, cells were washed 5 times with DMEM and cultured in DMEM/glutamine and 10% FBS or DMEM/glutamine or washed with DMEM/glutamine and 2% lipid supplement (chemically defined lipid concentrate containing fatty acids and cholesterol, Thermo Fisher Scientific Inc., Waltham, MA, USA, #11905031) for another 24 h.

### 2.3. Antibodies

The following antibodies were used: EPB41L5 (HPA037564, Atlas Antibodies, Bromma, Sweden, 1:300), NPHS1 (GP-N2, PROGEN Biotechnik GmbH, Heidelberg, Germany, 1:400), WT1 (ab15249, Abcam, Berlin, Germany, 1:400), desmin (M0760, Dako—Agilent Technologies, Germany GmbH & Co. KG, Waldbronn, Germany, 1:300), FHL2 (HPA006028, Atlas Antibodies, 1:300), CD3ε (#99940, Cell Signaling, 1:100–1:150), CD4 (#25229, Cell Signaling, 1:100–1:150), CD8α (#12653, Cell Signaling, 1:100–1:150), F4/80 (#70076, Cell Signaling, 1:200), CD19 (#90176, Cell Signaling, 1:100–1:300), CD11c (#97585, Cell Signaling, 1:100–1:300), PCNA (M0879, Dako, 1:100–1:600), ARG1 #893668S, Cell Signaling, 1:100), iNOS (#13120S, Cell Signaling, 1:150), phospho S6 ribosomal protein (Ser235/236) (#2211, Cell Signaling, 1:100–1:200), beta-actin (A5441, Merck/ Sigma-Aldrich Chemie GmbH, Taufkirchen, Germany, 1:200) and TER119 (#14-5921-82, Thermo Fisher Scientific, 1:300). Alexa Fluor secondary antibodies were purchased from Thermo Fisher Scientific, and HRP-linked secondary antibodies from Dako—Agilent Technologies and Merck/Sigma-Aldrich.

The antibodies used in this study are described in detail in supplemental Table S1 and protocols in respective methods sections.

### 2.4. SEM and TEM Procedures

Preparation of kidney samples for transmission electron microscopy (TEM) and scanning electron microscopy (SEM) was performed as previously described [15,24]. In brief, mice were sacrificed, and kidneys were perfused with 4% PFA in PBS via A. renalis. For TEM, small pieces of the renal cortex were dissected, and cubes of about 2 × 2 × 2 mm were cut using razor blades, followed by immersion fixation using 4% PFA and 1% GA in PBS for 24 h at 4 °C. Samples were stored in 0.1 M sodium cacodylate buffer at 4 °C until analysis. TEM tissue was post-fixed in 0.5% osmium tetroxide in ddH_2_O for 60 min on ice and washed 6 times in ddH_2_O. The tissue was incubated in 1% uranyl acetate in ddH_2_O at room temperature for 2 h. Dehydration was performed by 15 min incubations in increasing concentrations of EtOH and aceton. After embedding them in Durcupan resin, ultrathin sections were created using a UC7 Ultramicrotome (Leica Microsystems GmbH, Wetzlar, Germany) and collected on Formvar-coated copper grids. Imaging was done using a Zeiss Leo 912 transmission electron microscope. Embedding, semi-thin sectioning and electron microscopy were performed at the EM core facility of the Department of Nephrology, Faculty of Medicine, University of Freiburg. TEM images were analyzed for foot process (FP) width and GBM thickness (FP to endothelial cell distance) using FIJI ImageJ v1.52, as previously described [25].

For SEM, small, 5 × 5 × 5 mm samples were prepared and immersion fixated in 4% PFA and 1% GA in PBS for 3 days. Further dehydration was done in ethanol (50–100%—each 10% step for 1 h at RT) following transfer to hexamethyldisilazan (HMDS) (Sigma-Aldrich). Sputtering with gold was performed using a Polaron Cool Sputter Coater E 5100. Samples were visualized using a scanning electron microscope (Leo 1450 VP scanning). SEM was performed at the EM core facility of the Department of Nephrology, Faculty of Medicine, University of Freiburg.

### 2.5. Histology, IF and IHC Staining

Preparation of tissue samples, formalin fixation, paraffin embedding and microtome sectioning were performed using standard procedures at the Institute of Surgical Pathology, Faculty of Medicine, University of Freiburg. In brief, mice were sacrificed and organs were immersion fixed using 4% PFA in PBS for 24 h, at 4 °C. Kidneys were perfusion fixed with 4% PFA in PBS via A. renalis followed by immersion fixation for 24 h at 4 °C. For analysis of bone marrow sections, mice femur bone was decalcified in EDTA solution before paraffin embedding.

For immunofluorescence (IF) staining, 2 µm sections of formaldehyde-fixed, paraffin-embedded (FFPE) tissue were generated, deparaffinized and rehydrated and underwent heat-induced antigen retrieval (antibody-specific HIAR is described in supplemental Table S1). Sections were subsequently blocked in 5% BSA in PBS for 1 h, followed by incubation with primary antibodies in blocking solution for 2 h. Sections were washed 3 times in PBS and secondary fluorophore-tagged antibodies (Alexa Fluor Dyes, Thermo Fisher Scientific, Inc.) were applied in blocking solution for 45 min. Nuclei were stained using Hoechst 33342 (Thermo Fisher Scientific, Inc.). Sections were mounted in ProLong Gold Antifade (Thermo Fisher Scientific, Inc.) after repetitive washing in PBS. 

For immunohistochemistry (IHC), samples were sectioned and processed for primary antibody incubation as described for IF staining. In addition, peroxidase blocking was performed for 15 min using 1% H_2_O_2_ after HIAR. HRP-linked secondary antibodies (Dako—Agilent Technologies) were incubated in blocking solution on sections for 30 min. After repetitive washing in PBS, sections were stained using the DAB+ Substrate Chromogen System (Dako-Agilent Technologies), counter stained with Hematoxylin and dehydrated and mounted using Entellan. Alternatively to DAB, the Alkaline Phosphatase/RED (K5005, Dako—Agilent Technologies) staining system was used according to the manufacturer’s instructions.

Histology of FFPE tissue sections was performed from deparaffinized sections stained by periodic acid Schiff reaction (PAS), hematoxylin–eosin (HE), specific esterase and naphthol AS-D chloroacetate esterase (NACE) as well as acid fuchsin, orange-G and aniline blue (SFOG) using standard diagnostic staining procedures at the Department of Pathology, Faculty of Medicine, University of Freiburg.

For Oil Red O staining, 4 µm cryotome sections from unfixed, O.C.T. compound (Sakura Finetek Germany GmbH, Umkirch, Germany)-embedded tissue were used. Cryopreserved tissue was stored at −80 °C until use. Oil Red O (O0625, Merck/Sigma-Aldrich) staining was performed as described before [26].

### 2.6. IF Staining of Cultured Cells

For immunofluorescence (IF) staining of MΦs, cells were fixed in 4% PFA in PBS for 15 min. After fixation, cells were washed 3 times in PBS and permeabilized by 0.1% Triton X-100 in PBS for 3 min, followed by heat-induced antigen retrieval (HIAR) in pH 9.0 Tris-EDTA buffer at 90 °C for 40 min. Cells were washed in PBS and subsequently blocked in 5% BSA in PBS for 1 h at room temperature, followed by overnight incubation with primary antibodies in blocking solution at 4 °C (see supplemental Table S1). Samples were washed 3 times in PBS, and secondary, fluorophore-tagged antibodies (Alexa Fluor Dyes, Thermo Fisher Scientific, Inc.) were applied in blocking solution for 60 min. Nuclei were stained using Hoechst 33342 (Thermo Fisher Scientific, Inc.). Cells were washed 6 times with PBS and imaged in PBS. QuPath v0.2.1 software was used for cell segmentation and thresholding of positive cells as described below. Three independent replicates and at least 1469 cells per replicate and treatment condition were analyzed.

### 2.7. Iterative Indirect Immunofluorescence Imaging

Iterative indirect immunofluorescence imaging (4i) is a multiplex imaging technique, recently developed by Gut et al. for in vitro samples [27]. We adapted this technique for multiplex staining of 2 µm FFPE sections, following the published protocol. In brief, FFPE tissue was processed for IF staining, as described above. HIAR was performed in Tris-EDTA buffer at pH 9.0 using a pressure cooker for 10 min. After HIAR, a staining chamber was attached to the slide. All following buffers were prepared and applied as published by Gut et al.: conventional blocking solution (1% BSA in PBS), 4i blocking solution (1% BSA and 150 mM maleimide in PBS), elution buffer (0.5M L-glycine, 3M urea, 3M guanidinium chloride and 70mM TCEP-HCl in ddH_2_O, pH 2.5) and imaging buffer (700 mM N-acetyl-cysteine in in ddH_2_O, pH 7.4). Sections were washed 6 times with ddH2O, and elution buffer was applied 3 times for 10 min. Elution buffer was removed, and 4i blocking solution was added for 1 h. Samples were washed 6 times with PBS, and primary antibodies in conventional blocking solution were incubated for 2 h. Samples were washed 6 times with PBS, and secondary antibodies and Hoechst 33342, diluted in conventional blocking solution, were incubated for 45 min. Thereafter, samples were washed 6 times with PBS and 1 time with ddH_2_O. Imaging buffer was added, and defined regions of interest were imaged, as described in the next paragraph, under microscopy. Slides were stored in imaging buffer overnight at 4 °C. All other steps were performed at room temperature. These procedures were repeated for each antibody until required IF-plexity for the sample was reached. Image registration was performed using the Fiji ImageJ v1.52 descriptor-based registration plugin and Hoechst 33342 staining for registration. After registration, fluorescence intensities were manually adjusted for proper labeling of positive cells (excluding nonspecific background and auto-fluorescence signals) and QuPath image analysis software was used for cell segmentation and image analysis as described under histological analysis [28]. 

### 2.8. Microscopy

Immunofluorescence stainings were analyzed and imaged with an inverted Zeiss Axio Observer microscope (ApoTome.2, Axiocam 702 mono camera, Colibri 7 illumination system, 100×, 63×, 40×, 20× and 10× objectives and 49 DAPI, 38 GFP, 43 HE dsRed, 50 Cy5 filter sets) (Carl Zeiss AG, Oberkochen, Germany). For iterative indirect immunofluorescence imaging (4i), Hoechst 33342 stinging of cell nuclei was used for manual positioning of sample regions and repetitive imaging. In addition, an inverted Zeiss Axio Imager microscope equipped with an Axiocam color was used for analysis of IHC and histology. Stained sections were digitalized for analysis using a Ventana DP 200 slide scanner (Roche Diagnostics Deutschland GmbH, Mannheim, Germany) equipped with a 20× and a 40× objective.

### 2.9. Quantification of Glomerular Sclerosis

Glomerular sclerosis (GS) was scored using the 5-tier score [0–4] described before [29]. At least 50 glomeruli per animal were analyzed, and mean GS score per animal was calculated.

### 2.10. Quantification of Glomerular Podocytes

Kidney sections were IF stained for NPHS1 and WT1 as described above. Glomerular cells with positivity for NPHS1 and WT1 were identified as podocytes. In addition, tuft areas were measured using FIJI ImageJ v1.52 and finally, podocyte number per tuft area and glomerulus were calculated. At least 20 glomeruli per animal were analyzed.

### 2.11. Histological Analysis

QuPath v0.2.1 (https://qupath.github.io/, accessed 14 August 2020) image analysis software was used for quantitative analysis of FFPE sections [28]. For analysis of IHC stainings from WT and KO spleens, whole slides were digitalized using a Ventana DP 200 slide scanner. The QuPath built-in cell detection tool was used to segment individual cells from whole tissue sections based on hematoxylin nuclear staining. Individual thresholds for mean cellular staining intensity (or mean nuclear staining intensity, for PCNA and FOXP3) were defined for each staining, respectively, to classify cells as positive or negative. These classifiers were used to calculate positive cell fractions for respective WT and KO tissue sections. For segmentation of cell clusters from HE stained spleens, pixel classifiers were trained using the built-in neuronal network (ANN-MLP) to segment cellular dense (mainly white pulp) and sparse (mainly red pulp) tissue regions. Segmented objects with a size of <600 µm^2^ were excluded from analysis. Morphometric features of these segmented, dense cell clusters were analyzed. In addition, the Delaunay clustering plugin with a distance threshold of 400 µm from object centroids was used to analyze clustering/networks of dense cellular objects. For analysis of hepatocyte nuclear size, the QuPath cell detection tool was used to segment nuclei and measure nuclear areas from whole HE-stained liver sections. A minimum nuclear cutoff of 40 µm^2^ was used to exclude the majority of non-hepatocyte cell populations.

Analysis of 4i multiplex-IF images was performed in a similar way. After image registration, exemplary images were imported into QuPath for analysis. Cells were segmented using the built-in cell detection tool based on Hoechst 33,342 nuclear staining. Individual thresholds for mean cellular staining intensity (or mean nuclear staining intensity, for PCNA) were manually defined for each staining to classify cells as positive or negative. These single-channel classifiers were combined to composite classifiers to detect fractions of pS6 and PCNA single or double positive cells for respective immune cell marker positive cells (e.g., a classifier was combined to threshold for F4/80, pS6 and PCNA positivity, and the fractions of F4/80+ PCNA+ pS6+, F4/80+ PCNA- pS6+, F4/80+ PCNA+ pS6- and F4/80+ PCNA- pS6 cells were calculated).

### 2.12. Measurement of Urinary Albumin and Creatinine

Functionality of the glomerular filtration barrier was assessed by measurement of urinary protein levels (proteinuria), expressed as the albumin to creatinine ratio (ACR). The ACR was quantified by measuring spot urine from WT and KO mice at defined time points. Assessment of urinary albumin was performed using a mouse-specific albumin ELISA kit (ab108792, Abcam). Urinary creatinine measurement was performed using an enzymatic creatinine kit (Creatinine PAP LT-SYS LT-CR 0106, Labor & Technik, Eberhard Lehmann GmbH, Berlin, Germany). Assays were used according to the manufacturer´s instructions.

### 2.13. Gel Analysis

SDS-PAGE was performed using standard procedures. Urine samples were balanced according to creatinine concentration before analysis. Samples were denatured for 5 min at 95 °C by adding 2 × Laemmli buffer with DTT and loaded to a 4–15% precast polyacrylamide gel (Bio-Rad). Coomassie staining of gels was performed using a ready-to-use solution, according to the manufacturer´s instructions (Imperial Protein Stain, Thermo Fisher Scientific, Inc.). Bovine serum albumin standard ampules were used for generation of a standard curve (ThermoFisher Scientific, Inc.).

### 2.14. Analysis of Murine Blood Serum

Blood samples were collected by puncturing of the V. maxillaris of anesthetized mice. After clotting, blood samples were centrifuged at 2000× *g* for 10 min. Resulting supernatant (serum) was transferred into clean tubes and stored at −80 °C until use. Measurement of serum albumin was performed using a mouse-specific albumin ELISA kit (ab108792, Abcam). Creatinine and urea measurements were performed using enzymatic kits (Creatinine PAP LT-SYS LT-CR 0106, Urea LT-UR 0010, Labor & Technik, Eberhard Lehmann GmbH). Serum triglyceride (TG) levels were assayed using a fluorometric kit (ab178780, Abcam). Total cholesterol (TC), high-density lipoprotein (HDL), very low-density lipoprotein (VLDL) and low-density lipoprotein (LDL) fractions were measured using a calorimetric kit (ab65390, Abcam). Endotoxin (LPS) levels were determined using a chromogenic endotoxin quantification kit (A39552S, Thermo Fisher Scientific, Inc.). All assays were used according to the manufacturer´s instructions.

### 2.15. Microbiome Analysis

Feces samples from female WT and KO mice were collected from colorectal segments of the intestinal tracts from sacrificed mice. KO and control (“WT”) mice received doxycycline in parallel for 7 days at an age of 6 weeks. Feces samples were collected at least 8 weeks after completed doxycycline applications. Feces samples were snap frozen in liquid nitrogen and stored at −80 °C until use. The QIAamp Fast DNA Stool Mini Kit (51604, QIAGEN GmbH, Hilden, Germany) was used for preparation of bacterial DNA from stool samples. Quality control, generation of amplicon-based libraries and microbiome sequencing and profiling were performed by Eurofins Genomics Europe Sequencing GmbH, Konstanz, Germany. The Illumina MiSeq Personal Sequencer platform with MiSeq Reagent Kit v3 was used to generate 2 × 300 bp paired-end sequences of the bacterial 16S rRNA V3–V5 target region. Data processing was also performed by Eurofins Genomics. In brief, reads with ambiguous bases (“N”) and chimeric reads were removed based on the de-novo algorithm of UCHIME implemented in the VSEARCH package [30]. The remaining reads were processed into operational taxonomic units (OTUs) using minimum entropy decomposition (MED) [31]. DC-MEGABLAST alignments of cluster-representative sequences were performed to assign taxonomic information to each OTU. As a reference, database/dbdir/nt.ltered.fa (Release 13 February 2020) was used. OTUs and taxonomic assignments were further processed using the QIIME software package (version 1.9.1, http://qiime.org/, last accessed 11 June 2021) [32]. Abundances of bacterial taxonomic units were normalized as described by Angly et al. [33]. Principal component analysis (PCA) was performed using abundances of taxonomic units with ClustVis [34]. OTU and sample diversity was measured by calculation of the Shannon index, using Calypso [35]. Heat maps and statistics (two-way ANOVA with Sidak’s multiple comparisons test) were generated using GraphPad Prism 8 (GraphPad Software, San Diego, CA, USA) by analyzing the relative fraction of abundance-corrected reads assigned to respective taxonomic units. See also supplemental Dataset S1 for results of the microbiome analysis.

### 2.16. Quantification and Statistical Analyses

GraphPad Prism 8 software was used for statistical analyses and generation of graphs. Data are expressed as mean  ±  s.e.m. Scatter dots indicate the individual data points (samples, animals, etc.) used for statistical analysis. Unpaired t-tests, unpaired t-tests with Welch’s correction, the Mann–Whitney U test, one-way ANOVA with Tukey’s multiple comparisons test or two-way ANOVA with Sidak’s multiple comparisons test were used based on data distribution and experimental design. Data for urea, creatinine and OTU diversity were log transformed for statistical testing. PCA analysis was performed using ClustVis [34]. Statistical significance was defined as * *p*  <  0.05, ** *p*  <  0.01, *** *p*  <  0.001, **** *p*  <  0.0001 and n. s.—not significant. The number of independent experiments and total amounts of analyzed animals/samples are stated in the figures and/or figure legends.

## 3. Results

### 3.1. Inducible, Podocyte-Specific Knockout of Epb41l5 Results in FSGS Manifestation and Nephrotic Syndrome in Adult Mice

**Figure 1:**
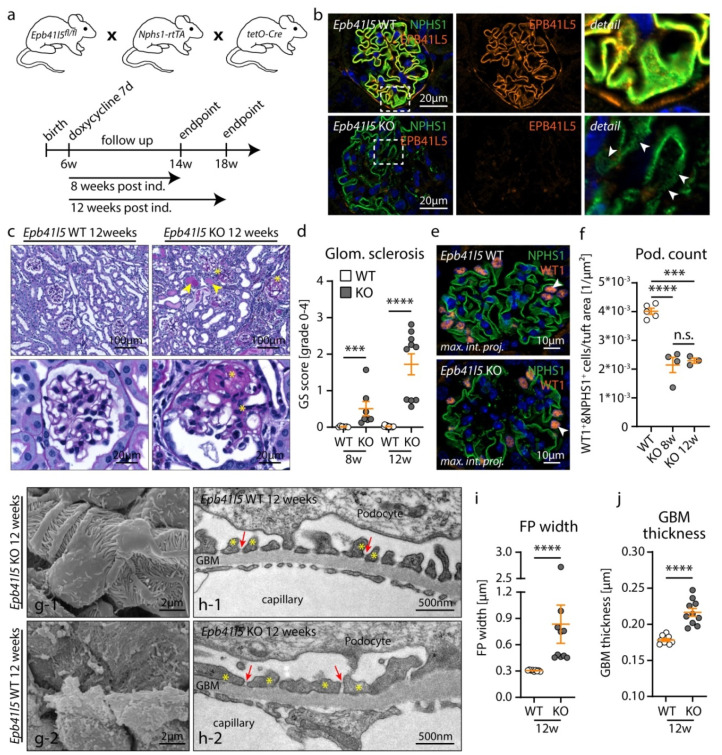
Inducible, podocyte–specific knockout of *Epb41l5* resulted in FSGS–like glomerulosclerosis. (**a**) Schematic illustrating the generation of a doxycycline–inducible, podocyte–specific *Epb41l5* knockout mouse model using the Epb41l5^fl/fl^*Nphs1–rtTA-–3G*tetOCre transgene. (**b**) Immunofluorescence analysis confirmed loss of EPB41L5 in podocytes 4 weeks p. i. The slit diaphragm (SD) component nephrin (NPHS1) was co–stained as a marker for the podocyte compartment. Reduced NPHS1 expression and widened foot process (FP) architecture (white arrowheads) were already detected 4 weeks p. i. (**c**) Loss of *Epb41l5* led to FSGS morphology accompanied by proteinaceous casts and tubular dilation (yellow arrows indicate proteinaceous casts, yellow asterisks mark glomerular sclerosis). (**d**) Quantification of glomerular sclerosis (GS) in *Epb41l5* control and knockout mice 8 and 12 weeks p. i. (dots indicate individual animals; *** *p* < 0.001, **** *p* < 0.0001). (**e**,**f**) Immunofluorescence staining for WT1 and NPHS1 demonstrated podocyte depletion from the glomerular basement membrane (GBM) (data of two 8 and three 12 weeks p. i. mice were pooled for statistical analysis of WT mice; *** *p* < 0.001, **** *p* < 0.0001). (**g**) Scanning electron microscopy of glomeruli demonstrated loss of FP architecture and podocyte detachment from the GBM in KO mice 12 weeks p. i.. (**h**–**j**) Transmission electron microscopy demonstrated pronounced widening (FP effacement) of podocyte FPs (yellow asterisks) in KO mice at 12 weeks p. i. SD density was decreased, and SDs (red arrows) were partially translocated to proximal parts of FPs. The GBM was thickened in KO mice (9 WT and 10 KO glomeruli from 3 WT and 4 KO animals were analyzed for mean FP width and GBM thickness; dots indicate individual glomeruli; **** *p* < 0.0001). Data are represented as mean ± SEM.

Glomerular disease with NS is a complex systemic disorder. However, studying NS in animal models is challenging due to the limitations of commonly-employed model systems. Loss of EPB41L5 results in progressive glomerulopathy with NS in a previously described, podocyte-specific knockout mouse model (Epb41l5^fl/fl^*hNPHS2Cre) with congenital onset [21,25]. We reasoned that controlled deletion of *Epb41l5* in adult mice (after completion of postnatal renal maturation) could model NS without common limitations of other reported experimental systems such as premature or prolonged disease onset and overlapping CKD phenotypes [15,24]. Here, we utilized the podocyte-specific Nphs1-rtTA-3G*tetOCre transgene to generate doxycycline-inducible Epb41l5^fl/fl^*Nphs1-rtTA-3G*tetOCre knockout mice (Figure 1a). To minimize phenotype interference by overlapping systemic CKD, respective experimental animals were sacrificed for analysis at 8 and 12 weeks post induction (p. i.). Initial analysis of Epb41l5^fl/fl^*Nphs1-rtTA-3G*tetOCre knockout mice confirmed highly efficient deletion of EPB41L5 expression. Moreover, altered and reduced expression of the slit diaphragm (SD) component nephrin (NPHS1) was observed already at 4 weeks after doxycycline induction (Figure 1b). Comprehensive morphological assessment of glomeruli using histology, immunofluorescence, scanning and transmission electron microscopy demonstrated successive development of the hallmark features of FSGS at 8 and 12 weeks p. i. (Figure 1c–j and supplemental Figure S1). Knockout mice exhibited progressive glomerular sclerosis, mesangial expansion and podocyte depletion from the GBM. Moreover, ultrastructural analysis confirmed podocyte foot process (FP) retraction (FP effacement), decreased SD density and GBM thickening.

**Figure 2:**
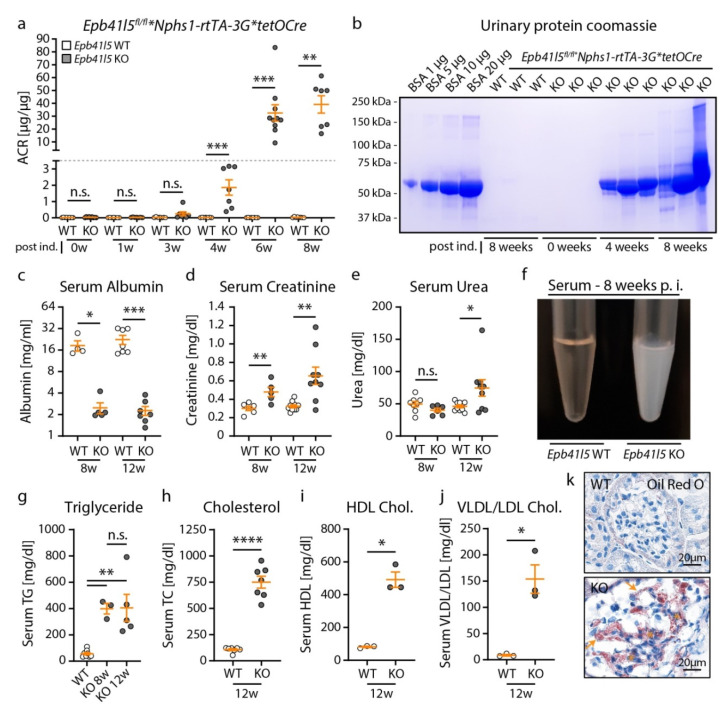
*Epb41l5* knockout mice developed severe nephrotic syndrome. (**a**,**b**) Progressive albumin and proteinuria were observed in adult Epb41l5^fl/fl^*Nphs1-rtTA-3G*tetOCre KO mice with an onset 3–4 weeks p. i. (ACR: urinary albumin to creatinine ratio). (**c**) Severe hypoalbuminemia was detected in KO mice already 8 weeks p. i.. (**d**,**e**) Serum creatinine and urea levels were only slightly elevated in KO mice 8 and 12 weeks p. i.. (**f**–**j**) KO mice developed marked hyperlipidemia characterized by strongly elevated levels of serum triglycerides (TG), total cholesterol (TC), HDL and VLDL/LDL (for 8 and 12 weeks p. i. time points data from WT animals were pooled for statistical analysis of TG). (**k**) Oil Red O staining of frozen kidney sections demonstrated lipid accumulation in glomeruli of KO mice. (**a**–**j**) Scatter dot plots indicate individual animals (n.s.: not significant, * *p* < 0.05, ** *p* < 0.01, *** *p* < 0.001, **** *p* < 0.0001). Data are represented as mean ± SEM.

Urine protein levels were analyzed to determine glomerular disease onset at different time points (Figure 2a,b), and progressive proteinuria was detected in Epb41l5^fl/fl^*Nphs1-rtTA-3G*tetOCre knockout mice, with an onset of albuminuria at 3–4 weeks p. i. KO animals showed severe hypoalbuminemia, detected by analysis of serum albumin levels already 8 weeks p. i. (Figure 2c). Interestingly, serum creatinine and urea levels were only minimally increased, substantiating our attempt to establish a NS model with the slowly progressive renal failure characteristic of many human conditions associated with NS (Figure 2d,e). Serum from KO mice showed obvious lipidic coloration (Figure 2f), and further detailed analysis demonstrated significantly elevated triglycerides, total cholesterol, HDL and VLDL/LDL levels in sera from KO mice (Figure 2g–j). Moreover, Oil Red O staining of frozen kidney sections showed glomerular accumulation of lipid droplets in knockout mice (Figure 2k). 

Collectively, Epb41l5^fl/fl^*Nphs1-rtTA-3G*tetOCre mice developed typical FSGS-like glomerular disease with hallmark features of severe NS but exhibited only minimal overlapping CKD phenotypes.

### 3.2. Nephrotic Syndrome Was Accompanied by Alterations of the Microbiome Composition

**Figure 3:**
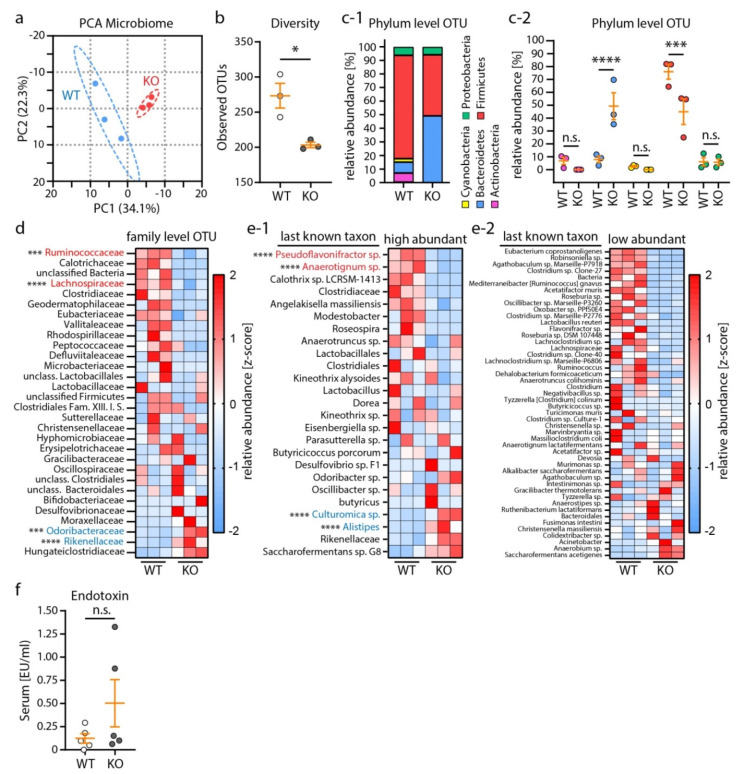
Nephrotic syndrome altered microbiome composition in mice. (**a**) Principle component analysis (PCA) of microbiome samples from 3 WT and 3 KO mice (95% prediction ellipses are shown). (**b**) Microbiome sample diversity expressed as the Shannon index, calculated from detected operational taxonomic units (OTUs). (**c**) Analysis of OTUs on the phylum level revealed increased relative abundance of Bacteroidetes OTUs and reduced relative abundance of Firmicutes OTUs. (**d**,**e**) Heatmaps of z–scores for relative abundance of family level OTUs (**d**) or last known taxon level OTUs (**e**) revealed specific alterations of the microbiome composition in nephrotic mice. Red color–coded OTUs indicate significant regulated Firmicutes taxa. Blue color–coded OTUs indicate significant regulated Bacteroidetes taxa. Significance levels were only indicated for these taxa. (**f**) Measurement of serum endotoxin levels (EU: endotoxin units). (**a**–**f**) Scatter dot plots indicate individual animals (n.s.: not significant, * *p* < 0.05, *** *p* < 0.001, **** *p* < 0.0001). Data are represented as mean ± SEM.

Recent studies reported distinct alterations of the gut microbiome in human CKD patients and mouse models with CKD features [36,37,38,39,40,41,42]. Moreover, dysbiosis of the gut microbiome has been recently reported in patients suffering from NS [43,44]. Therefore, we mapped the gut microbiome in our model by sequencing the 16S rRNA V3–V5 target region. Principle component analysis of stool samples from female mice revealed distinct changes in the microbiome of respective knockout mice (Figure 3a). 

General sample microbiome diversity was reduced, and the relative abundance of phylum-level operational taxonomic units (OTUs) was shifted from Firmicutes to Bacteroidetes OTUs in KO mice (Figure 3b,c). Detailed analysis of mapped taxa revealed a highly significant reduction of Pseudoflovonifractor sp. and Anaerotignum sp. as well as enrichment of Culturomica sp. and Alistipes OTUs, as part of underlying alterations (Figure 3d,e). Given this pronounced dysbiosis and based on previous reports from CKD models, we measured systemic endotoxin (LPS) levels (Figure 3f). However, we did not detect a significant increase in serum endotoxin levels, indicating no relevant intestinal barrier dysfunction or related systemic inflammatory processes in our model.

### 3.3. Nephrotic Syndrome Leads to Multiple Organ Pathologies in Mice, Including Reactive Hepatomegaly

**Figure 4:**
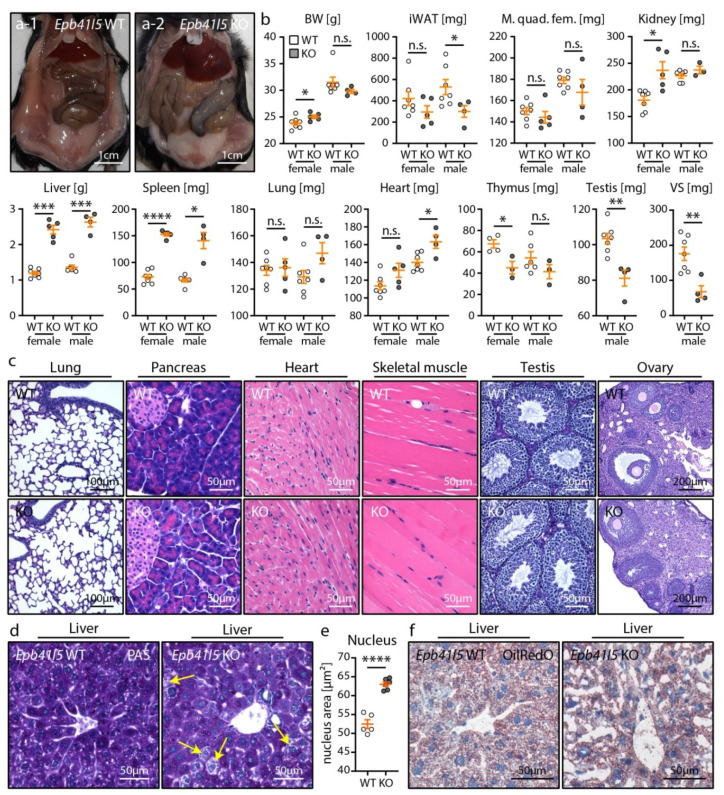
Nephrotic syndrome led to multiple organ pathologies in mice, including reactive hepatomegaly. (**a**) Abdominal situs of *Epb41l5* WT and KO mice 12 weeks p. i. demonstrated hepatomegaly. (**b**) Analysis of organ weights revealed multiple organ pathologies 12 weeks p. i. (vesicula seminalis (VS), inguinal white adipose tissue (iWAT)). (**c**) HE staining of representative organs showed no obvious microscopic pathologies. Gonads were still functional, as indicated by ovary follicle development and spermatozoa production in the testis (see also supplemental Figure S2 for histology of further organs). (**d**,**e**) PAS staining of liver sections revealed paled cytoplasm (yellow arrows) and enlarged nuclei of hepatocytes. Notably, no other signs of hepatopathy or fibrosis were detected (5 WT and 6 KO mice 12 weeks p. i. were analyzed). (**f**) Oil Red O staining of frozen liver sections showed no overall altered lipid levels in livers of nephrotic mice. (**a**–**e**) Scatter dot plots indicate individual animals (n.s.: not significant, * *p* < 0.05, ** *p* < 0.01, *** *p* < 0.001, **** *p* < 0.0001). Data are represented as mean ± SEM.

Next, we utilized our novel mouse model for the analysis of NS-associated organ pathologies in mice by detailed macroscopic and histological characterization at 12 weeks p. i. (Figure 4 and supplemental Figure S2). Analysis of organ weights and macroscopic appearance revealed multiple organ pathologies such as hepatomegaly and splenomegaly as well as hypotrophy of male gonads, vesicula seminalis (VS) and inguinal white adipose tissue (iWAT) in nephrotic mice (Figure 4a,b). Notably, features often attributed to progressed CKD (e.g., reduced body weight and muscle weight) were not significantly altered in knockout animals [45]. In line with this, decreased iWAT in male mice might have been related to impaired lipid and energy metabolism as well as excessive protein loss due to NS (Figure 2) [46]. Hypotrophy of the male reproductive tract could be explained by the loss of sex hormones and sex hormone-binding proteins into the urine, as observed by others [47,48]. However, histological analysis of female and male gonads indicated preserved physiological function, as ovary follicle development and spermatozoa production in the testis were not obviously impaired (Figure 4c). Histological analysis of further representative organs showed no major relevant histopathological abnormalities except for liver and spleen parenchyma (Figure 4c and supplemental Figure S2). PAS staining of liver tissue revealed paled cytoplasm and enlarged nuclei of hepatocytes (Figure 4d,e). Moreover, largely increased liver weight was already detected 8 weeks p. i. (supplemental Figure S2). Notably, no additional signs of hepatopathy such as fibrosis or extramedullary hematopoiesis were detected, and Oil Red O staining of frozen liver sections showed normal lipid (triglyceride) levels in the livers of nephrotic mice (Figure 4f). Therefore, this phenotype was possibly related to the observed severe hypoproteinemia, which might translate into reactive hepatic protein synthesis [48].

### 3.4. Nephrotic Syndrome Causes Extramedullary Hematopoiesis and Red Pulp Macrophage (RpMΦ) Expansion

**Figure 5:**
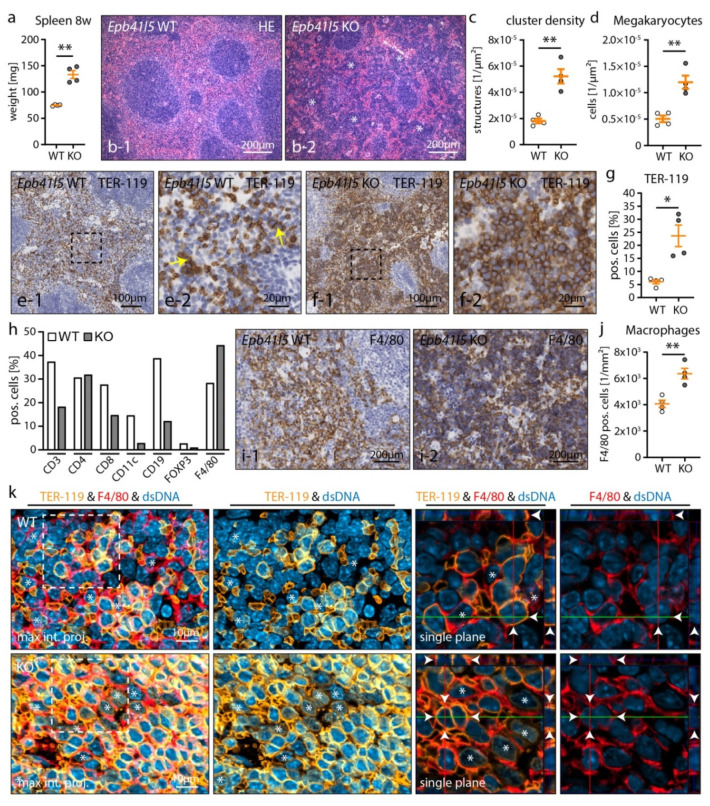
Nephrotic syndrome caused extramedullary hematopoiesis and RpMΦ expansion. (**a**) Nephrotic mice presented with splenomegaly already 8 weeks p. i. (**b**–**d**) HE analysis of spleen tissue showed increased appearance of dense cell clusters (white asterisks) and megakaryocytes in red pulp tissue, indicative for EMH (see also supplemental Figure S3). (**e**–**g**) IHC analysis of the erythroid lineage marker, TER–119, confirmed EMH in KO spleens. Erythrocytes, but only a few erythroid lineage cells (yellow arrows), were detected in the spleens of WT animals. (**h**) Screening for major immune cell populations revealed relative reduction of T and B lymphocytes and an increase of F4/80 positive red pulp macrophages (RpMΦ) (see also supplemental Figure S4). (**i**,**j**) IHC staining for F4/80 confirmed relative expansion of RpMΦs in spleens of nephrotic mice. (**k**) IF analysis for TER–119 and F4/80 demonstrated close interaction of erythroid cells and RpMΦs in the hematopoietic niche. Weak TER–119 positivity of RpMΦs in the spleens of KO mice suggested increased erythrophagocytosis by RpMΦs (white asterisks indicate RpMΦs, white arrowheads indicate adjacent cell membranes of TER–119 positive erythrocyte linage cells with F4/80 positive RpMΦs in orthogonal sections of z–stacks). (**a**–**k**) Mice were analyzed 8 weeks p. i.; scatter dot plots indicate individual animals (n.s.: not significant, * *p* < 0.05, ** *p* < 0.01). Data are represented as mean ± SEM.

The spleen exerts several homeostatic functions, ranging from immune response to erythrocyte filtering, in health and systemic diseases. In our model, nephrotic mice developed a significant increase in weight and size of the spleen at 12 and even 8 weeks post induction (Figure 4b and Figure 5a). We analyzed the spleen histoarchitecture of respective wild type and knockout mice 8 weeks p. i. to assess the impact of a global NS condition. Initial analysis revealed a marked disorganization of the spleen histoarchitecture, which is anatomically organized into white pulp (lymphoid follicles) and red pulp tissue (sinusoids of the blood filtering system and red pulp macrophages (RpMΦ)). These alterations were characterized by the expansion of compact cell clusters and megakaryocytes in the red pulp tissue—indicative for extramedullary hematopoiesis (EMH) of the spleen [49] (Figure 5b–d and supplemental Figure S3). 

Staining for the erythroid lineage marker, TER-119, confirmed the expansion of erythropoietic lineage cells and the formation of erythroblastic islands (EBIs) in the red pulp tissue of nephrotic mice (Figure 5e–g). In contrary to erythropoiesis and megakaryopoiesis, no signs for extramedullary granulopoiesis were detected by HE and chloroacetate esterase staining (supplemental Figure S3). Notably, no obvious decrease in medullary hematopoiesis was detectable, largely excluding suppressive bone marrow pathologies as a causative mechanism (supplemental Figure S3). Next, we screened for major immune cell populations of the spleen to further investigate the impact of EMH (Figure 5h and supplemental Figure S4). This analysis revealed a relative reduction of T and B lymphocyte populations and a concomitant increase of RpMΦ in the spleen (indicated by the expansion of F4/80-positive cells—Figure 5i,j). Of note, no shift from RpMΦ characteristic M1 macrophage polarization to M2 polarization was detected (supplemental Figure S4). While reduction of lymphocytes might be caused by relative displacement due to EMH, RpMΦ expansion has been described as an essential component of EBIs and hematopoietic niche formation during EMH in mice [50]. EBIs are structurally composed of central macrophages surrounded by immature erythroblasts. Functionally, erythroblast island macrophages (EBIMΦ) were identified as essential stimulators and regulators of EBI maturation [51,52,53]. In line with these previous observations, in our model RpMΦs/EBIMΦs were found as components of EBIs closely enwrapping erythroid cells (Figure 5k). Moreover, the RpMΦ of nephrotic but not of WT mice showed cytoplasmic positivity for the erythroid linage marker TER-119, indicative of increased hemophagocytosis by RpMΦ [54]. Taken together, nephrotic syndrome in mice induced prominent EMH of the spleen characterized by RpMΦ/EBIMΦ and erythroblast expansion and formation of EBIs.

### 3.5. Hematopoietic Niche Expansion Correlates to RpMΦ Proliferation and mTOR Activation

**Figure 6:**
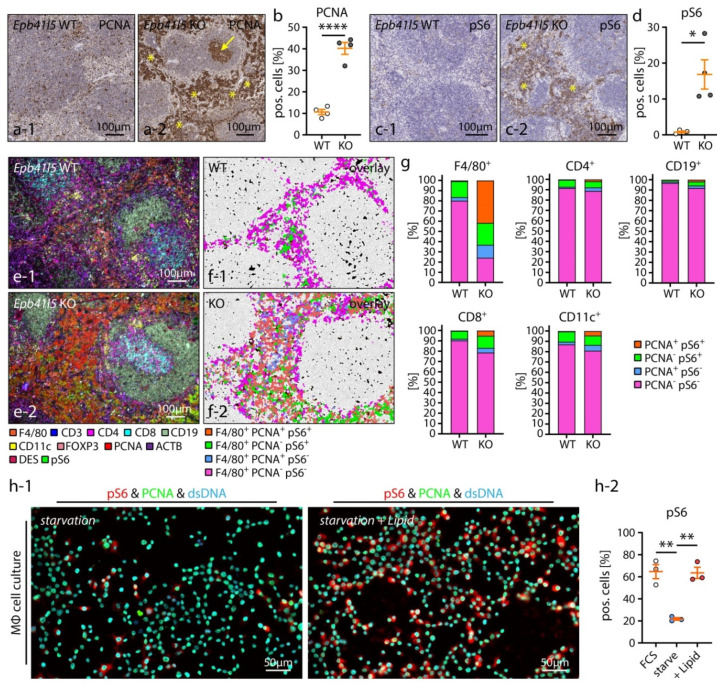
Hematopoietic niche expansion correlated to RpMΦ proliferation and mTOR activation. (**a**–**d**) Staining for proliferating cell nuclear antigen (PCNA) and phospho–S6 ribosomal protein (pS6) demonstrated a distinct increase of PCNA positive and pS6 positive cells in erythroblastic islands (EBIs) (yellow asterisks) and of PCNA in germinal centers (yellow arrows) of KO animals 8 weeks p. i. (scatter dot plots indicate individual animals; * *p* < 0.05, **** *p* < 0.0001). (**e**–**g**) Multiplex IF staining applying iterative indirect immunofluorescence imaging (4i) identified F4/80 positive RpMΦs as one source of PCNA positive and pS6 positive cells in the EBIs of KO animals. Subfigures (**e-1**) and (**e-2**) show multiplex IF images of murine spleens. Individual IF markers were color–coded as indicated. Subfigures (**f-1**) and (**f-2**) show overlays of segmented cells and color–coding of cells positive for F4/80 and/or–PCNA and/or pS6 as indicated. Subfigure (**g**) shows relative abundances of co–positivity for PCNA and/or pS6 for major immune cell populations of the spleen. (**h**) Cultured MΦs were starved or stimulated by a lipid mixture containing fatty acids and cholesterol or by fetal bovine serum (FCS). MΦs were stained for PCNA and pS6 and the percentage of pS6 positive cells was quantified (scatter dot plots indicate individual experiments; ** *p* < 0.01). Data are represented as mean ± SEM.

Next, we aimed to elucidate the underlying molecular mechanisms of EBI expansion. The mechanistic target of rapamycin (mTOR) controls essential cellular processes such as organ growth, cell proliferation and metabolism [55]. Moreover, mTOR signaling has been previously described as a critical regulator of erythropoiesis and MΦs [56,57]. We analyzed cell growth and proliferation in the spleen by staining for proliferating cell nuclear antigen (PCNA) and the mTOR downstream phosphorylation target S6 ribosomal protein (S6) (Figure 6a–d). Here, we observed a pronounced increase of PCNA and phospho-S6 (pS6) positive cells in the red pulp tissue and EBIs of respective nephrotic animals. To clarify the identity of these PCNA positive and pS6 positive cell populations, we utilized a recently described multiplex fluorescence imaging technique (4i, iterative indirect immunofluorescence imaging [27]) and adapted this technique for FFPE in situ samples (Figure 6e–g and supplemental Figure S4). Employing this multiparametric imaging approach, we confirmed F4/80 positive RpMΦs/EBIMΦs as a major source of the PCNA positive and pS6 positive cell population. This observation is indicative for growth stimulation and activation of EBIMΦs in nephrotic mice. Lipids such as cholesterol and fatty acids are stimulators of the mTOR signaling pathway [58,59,60]. Therefore, we hypothesized that increased serum lipid levels in NS might contribute to mTOR activation in MΦ [61]. In line with this hypothesis, lipid-stimulated MΦs showed significantly increased pS6 levels in cell culture compared to unstimulated cells (Figure 6h), indicative of a potential direct relation between NS and EBIMΦ activation in nephrotic mice.

## 4. Discussion

Nephrotic syndrome (NS) is a complex systemic disease with major implications for essential homeostatic functions and organ systems. Here, we described a novel, inducible Epb41l5^fl/fl^*Nphs1-rtTA-3G*tetOCre model for NS in adult mice (Figure 1). Podocyte-specific promotors (e.g., *Nphs1* and *Nphs2*) have been extensively employed to generate conditional knockout models based on the restricted expression pattern of respective genes. However, recombination in cell types other than podocytes cannot be completely excluded. By combination with a highly podocyte-selective and essential gene like *Epb41l5*, our model allowed for efficient gene deletion of *Epb41l5* in the podocytes of adult mice, resulting in podocyte depletion and the rapid onset of glomerular disease. Moreover, this model confirmed the essential role of EPB41L5 for podocyte function in fully matured glomeruli beyond our recently published Epb41l5^fl/fl^*hNPHS2 model with congenital onset of glomerular disease [21,25]. Respective transgenic mice developed FSGS-like glomerular sclerosis and hallmark characteristics of severe NS, including proteinuria, hypoalbuminemia and dyslipidemia (Figure 1 and Figure 2).

Several recent studies found distinct alterations of the gut microbiome in human and murine CKD [36,37,38,39,40,41,42]. Beyond this evident role in CKD, dysbiosis of the gut microbiome has been described in patients suffering from nephrotic syndrome [43,44]. However, knowledge concerning the role of the gut microbiome in murine NS conditions is rather limited [62]. Therefore, we used our novel mouse model to map the gut microbiome and found marked changes in microbiome composition in nephrotic mice (Figure 3). We detected a pronounced increase of the Bacteroidetes phylum and the Alistipes taxon as part of the underlying alterations. The Alistepes genus has been suggested to promote anti-inflammatory and hepatoprotective functions, potentially via the production of short chain fatty acids (SCFAs) [63]. This increase might, therefore, be part of an adaptive or protective response in NS. However, Bacteroidetes and Alistipes are potent producers of harmful metabolites including ammonia, hydrogen sulfide, cresol, indole and phenol [63,64]. Moreover, indole and cresol are metabolized to uremic toxins such as indoxyl sulfate and p-cresyl sulfate [37,65]. Intriguingly, indoxyl sulfate has been shown to initiate tubulointerstitial damage and promote CKD progression in a microbiome-dependent manner [37,38,66]. Therefore, NS-associated dysbiosis might be involved in the disease progression to CKD. The exact mode of NS-dependent microbiome alterations is multifactorial, involving features of NS-like hyperlipidemia, hypoproteinemia, dysregulation of hormone signaling and immune/inflammatory processes. Interestingly, intestinal tract edema is a well-described clinical complication of NS and an established driver for intestinal barrier and microbiome dysfunction in the context of heart failure [67,68,69]. Moreover, in our model, we observed severe hypoalbuminemia and mild ascites, factors suggestive of the presence of intestinal edema. Therefore, the interdependency of intestinal barrier edema, microbiome composition and barrier function might be a promising target for further research on NS.

Further characterization of organ pathologies revealed prominent splenomegaly and manifest extramedullary hematopoiesis (EMH) as underlying pathologies (Figure 4 and Figure 5). In principle, EMH most frequently occurs due to bone marrow failure, myelostimulation, inflammation or an altered chemokine microenvironment [70]. Anemia is a frequently observed complication of nephrotic syndrome but occurs as a result of urinary loss of erythrogenic factors such as iron, transferrin and erythropoietin [71,72,73]. Therefore, physiological stimulation of erythropoiesis is unlikely in NS. Interestingly, EMH in the spleen has been described before in the nephron toxic serum (NTS) model for glomerular disease [74]. Moreover, this study detected a concomitant anemia and repression of bone marrow hematopoiesis. Interestingly, a similar phenotype has been described in a model of systemic lupus erythematosus (SLE) [75]. Here, the authors speculated that the chronic systemic inflammatory status of NTS and SLE models might contribute to the observed bone marrow and EMH phenotypes. In contrast to those studies, we observed no major abnormalities in bone marrow hematopoiesis in our model (supplemental Figure S3). Our non-immunological/non-toxic murine model points to an NS-dependent mechanism leading to EMH in the spleen. Together, these observations argue against obvious bone marrow failure, the physiological stimulation of erythropoiesis or inflammation as the sole causative mechanism for EMH, suggesting alterations of the microenvironment of the hematopoietic niche as potentially contributing factors for EMH.

The erythroblastic island (EBI) is the functional unit of the hematopoietic niche, and EBIs are composed of a central macrophage surrounded by immature erythroblasts [76]. Functionally, erythroblast island macrophages (EBIMΦs) shape the EBI microenvironment, an essential process for EBI regulation [51,52,53]. Detailed analysis of EBIs revealed an activated and proliferative state of EBIMΦs in nephrotic mice (Figure 6), potentially driving EMH. Activation of RpMΦs/EBIMΦs might result from many contributing factors in NS such as cytokine [77,78] or microbiome alterations (Figure 3). Our observations and experiments even pinpoint a direct role of nephrotic dyslipidemia for macrophage activation and EMH propagation (Figure 6—[7,61]). Interestingly, ample evidence suggests a role of the spleen in lipid metabolism to prevent hyperlipidemia, probably involving LDL catabolism and lipid storage by MΦs [79]. Moreover, defects in cholesterol efflux pathways have been shown to lead to mobilization of hematopoietic stem and progenitor cells and to EMH of the spleen. This phenotype was mediated by the secretion of IL-23 from phagocytic cells of the spleen [80]. Elevated cholesterol levels and reduced cholesterol efflux in NS might therefore translate into activation of MΦs and EMH [7]. Direct implications of NS-associated EMH in mice for human disease conditions remain so far incomplete. Interestingly, there are single reports indicating an interrelationship between treatment-resistant NS conditions and the development of hemophagocytic lymphohistiocytosis caused by the activation of MΦs [81]. Moreover, EMH of the spleen was linked to propagation of atherosclerosis, potentially mediated by the activation of splenic MΦs and MΦ-dependent lipid regulation [82]. Therefore, further research evaluating the exact molecular mechanism of NS, MΦ activation and EMH might contribute to a better understanding of MΦ-associated pathologies (e.g., atherosclerotic vascular disease) in the context of NS. 

## 5. Conclusions

In summary, we described here a novel, inducible podocyte-specific model (Epb41l5^fl/fl^*Nphs1-rtTA-3G*tetOCre) for NS in adult mice. We utilized this model to analyze the impact of NS on an organ system-level, including detailed analysis of the microbiome composition and organ pathologies such as extramedullary hematopoiesis of the spleen, demonstrating the versatility of this model for nephrotic syndrome-related research. In a translational context, our model highlights the complex organotypic interdependencies in NS and indicates dysbiosis of the gut microbiome and activation of spleen MΦs as potentially relevant factors in NS. Therefore, further studies on animal models and human NS might add novel entities such as dysbiosis of the gut microbiome to the list of clinically relevant complications of NS, eventually leading to new therapeutic approaches.

## Data Availability

The data presented in this study are available in supplementary Dataset S1 and Dataset S2.

## Supplementary Materials

The following are available online at https://www.mdpi.com/article/10.3390/cells10061509/s1, Figure S1: Analysis of *Epb41l5* KO glomeruli, corresponding to Figure 1. Figure S2: Organ and tissue analysis, corresponding to Figure 4. Figure S3: Analysis of spleen EMH in NS, corresponding to Figure 5. Figure S4: Analysis of spleen immune cell populations in NS, corresponding to Figure 6. Table S1: Antibodies used in this study. Dataset S1: Microbiome analysis, corresponding to Figure 3. Dataset S2: OUT-table (biom file) of microbiome analysis, corresponding to Figure 3.
