# Supplementary material for: A Novel Model for Nephrotic Syndrome Reveals Associated Dysbiosis of the Gut Microbiome and Extramedullary Hematopoiesis
Source: Cells. 2021 Jun 15;10(6):1509. doi: 10.3390/cells10061509 (PMC8232754; doi:10.3390/cells10061509)
Supplement: Supplementary file 1 [file cells-10-01509-s001.zip › Supplemental Figures.pdf]

Supplemental Information

Figure S1

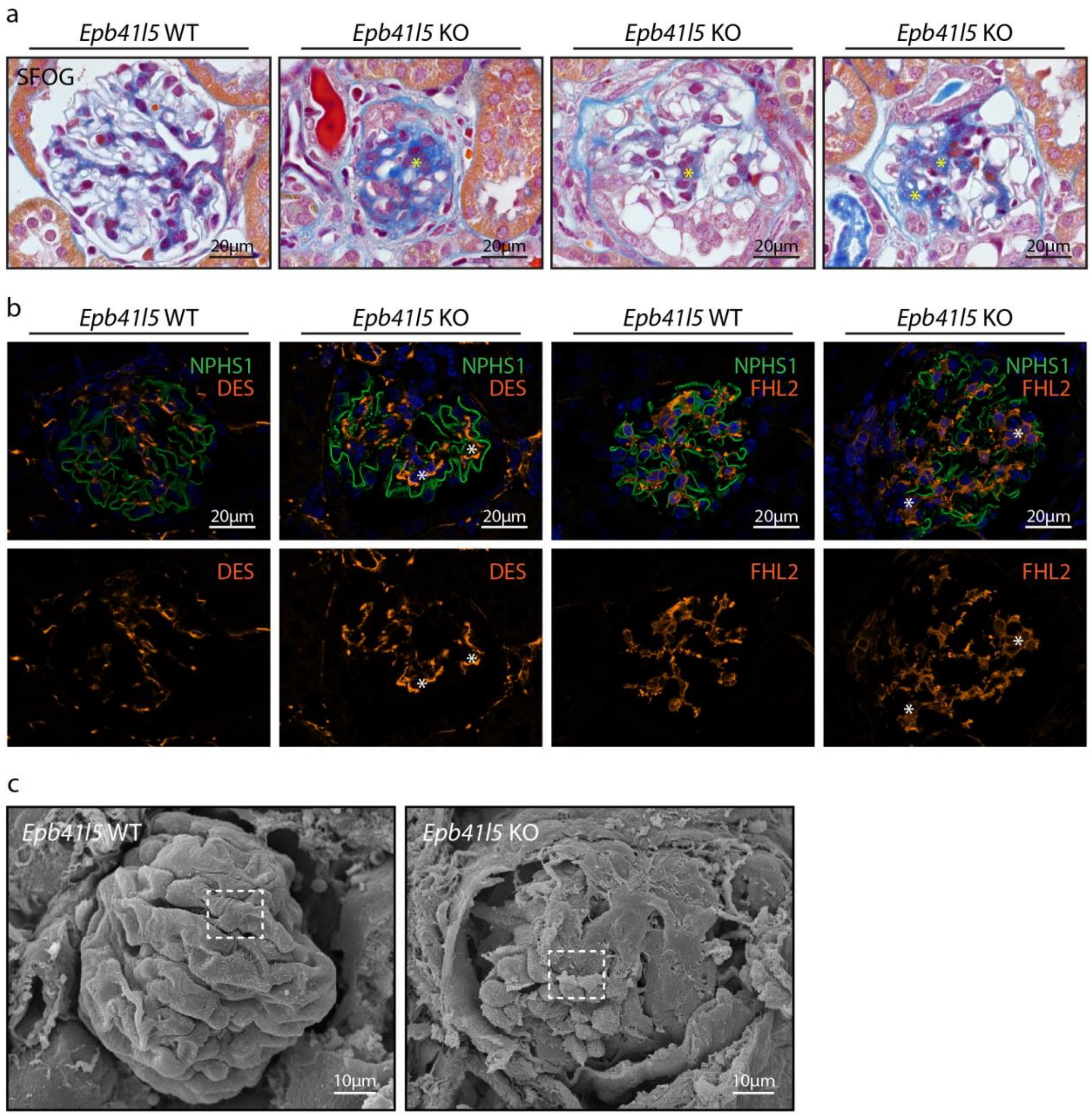

Supplemental Figure 1

(a) SFOG staining of WT and KO mice 12 weeks p. i. demonstrates glomerular fibrosis (yellow asterisks). Blue staining marks collagen filaments.

(b) IF staining for mesangial cells by Desmin (DES) and Four and a half LIM domains protein 2 (FHL2) demonstrates mesangial expansion (white asterisks) in KO mice 12 weeks p. i.. Nephrin (NHPS1) was used to stain the podocyte compartment.

(c) Scanning electron microscopy overview images of *Epb41l5* WT and KO glomeruli. Dashed line boxes indicate zoom in regions shown in main Figure 1.

Figure S2

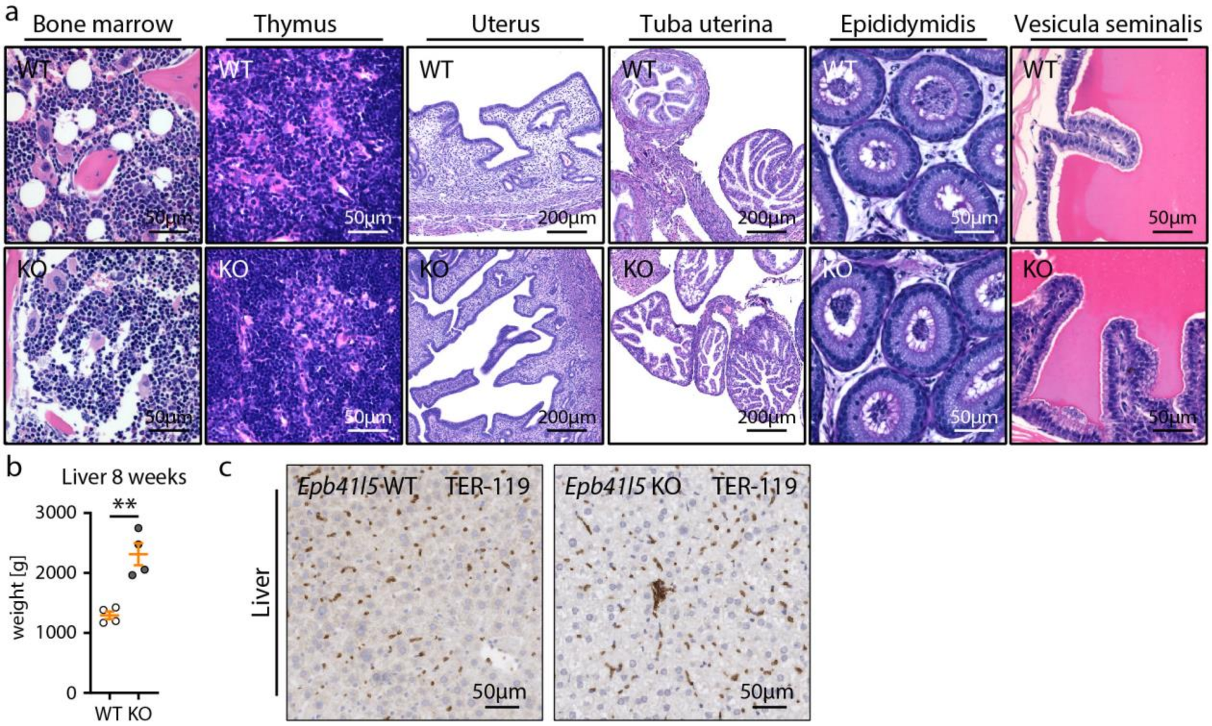

Supplemental Figure 2

(a) Representative HE stainings of organs from *Epb41l5* WT and KO mice 12 weeks p. i., reduction of bone marrow adipocytes was observed (analysis corresponding to main Figure 4). No further obvious microscopic pathologies were observed.

(b) Analysis of liver weights of *Epb41l5* WT and KO mice reveal hepatomegaly 8 weeks p. i. (** p<0.01; analysis corresponding to main Figure 4). Data are represented as mean ± SEM.

(c) IHC analysis of the erythroid lineage marker TER-119 in liver tissue of WT and KO animals. Erythrocytes but no erythroid lineage cells were detected by TER-119 staining.

Figure S3

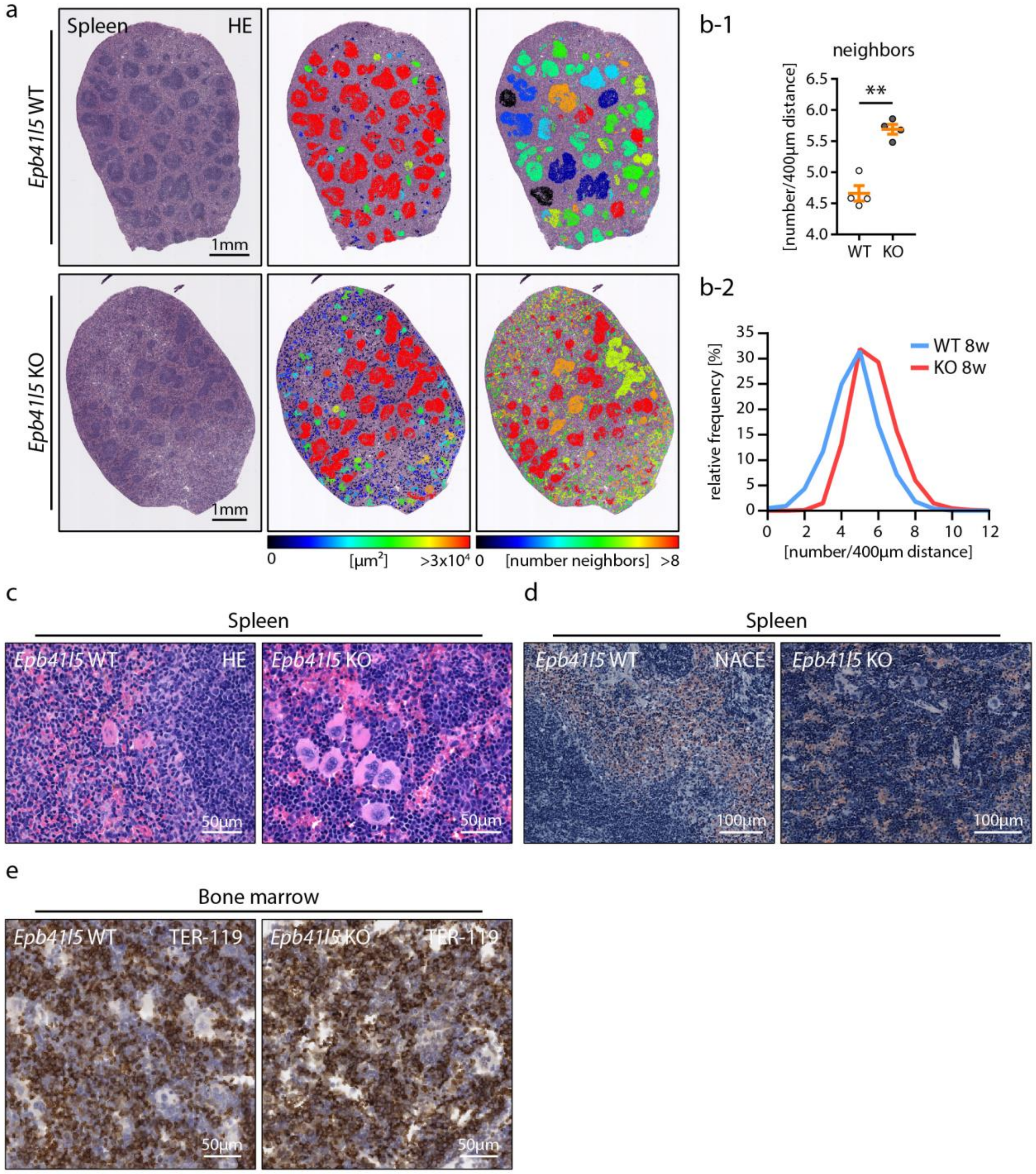

Supplemental Figure 3

(a) Representative HE stainings of spleen sections 8 weeks p. i. illustrate segmentation of dense cell clusters including white pulp tissue of the spleen using the QuPath software. Relative cluster size and number of neighboring clusters (<400µm distance of cluster centroids) were color-coded as indicated (analysis corresponding to main Figure 5).

(b) Number of neighboring clusters is increased in spleen of KO mice. Data are represented as mean ± SEM.

(c) Spleen of *Epb41l5* KO mice exhibit increased numbers of megakaryocytes 8 weeks p. i.. Representative HE stainings of WT and KO spleens are shown (images corresponding to quantification in main Figure 5).

(d) Specific esterase, naphthol AS-D chloroacetate esterase (NACE) staining shows no obvious expansion of neutrophil immune cell populations in spleens of KO mice 8 weeks p. i..

(e) IHC analysis of the erythroid lineage marker TER-119 in bone marrows of WT and KO animals 8 weeks p. i. shows no obvious difference in TER-119 positive cell population.

Figure S4

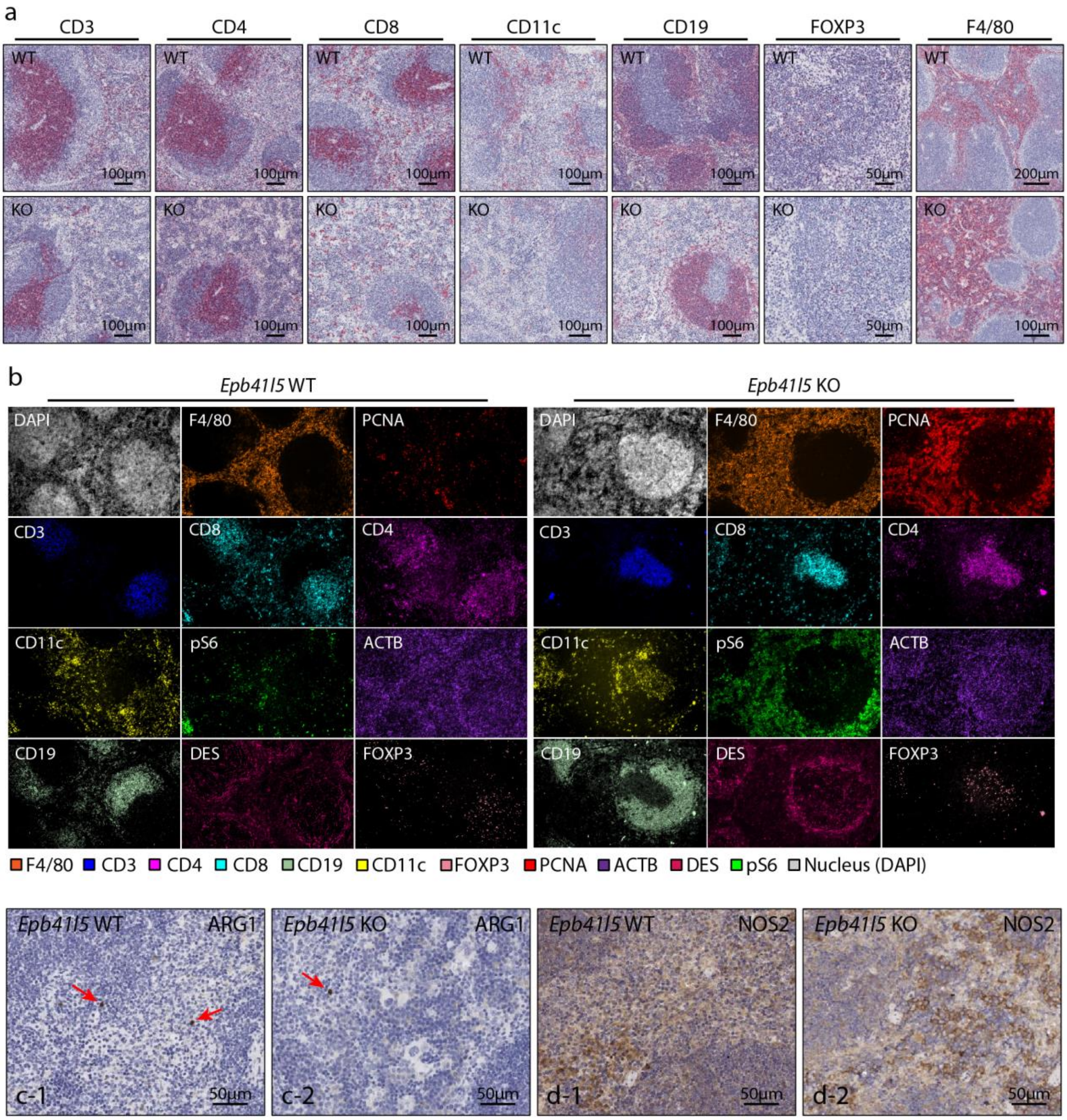

Supplemental Figure 4

(a) IHC staining for CD3, CD4, CD8, CD11c, CD19 and FOXP3 in WT and KO spleens 8 weeks p. i. (analysis corresponding to main Figure 5).

(b) Single channel IF images corresponding to multiplex 4i imaging shown in main Figure 6.

(c&d) IHC staining for Arginase-1 (ARG1) and iNOS (NOS2) show no expansion of ARG1 positive M2 macrophage population but confirmed stable expression of the M1 macrophage marker iNOS in spleen tissue of KO mice 8 weeks p. i..
